# Serum Amino Acids Profile and the Beneficial Effects of L-Arginine or L-Glutamine Supplementation in Dextran Sulfate Sodium Colitis

**DOI:** 10.1371/journal.pone.0088335

**Published:** 2014-02-05

**Authors:** Wenkai Ren, Jie Yin, Miaomiao Wu, Gang Liu, Guan Yang, Yan Xion, Dingding Su, Li Wu, Tiejun Li, Shuai Chen, Jielin Duan, Yulong Yin, Guoyao Wu

**Affiliations:** 1 Scientific Observing and Experimental Station of Animal Nutrition and Feed Science in South-Central, Ministry of Agriculture, Hunan Provincial Engineering Research Center of Healthy Livestock Key Laboratory of Agro-ecological Processes in Subtropical Region, Institute of Subtropical Agriculture, Chinese Academy of Sciences, Changsha, Hunan, China; 2 School of Food Science, Washington State University, Pullman, Washington, United States of America; 3 Department of Animal Science, Texas A&M University, College Station, Texas, United States of America; 4 Laboratory of Animal Fat Deposition and Muscle Development, College of Animal Science and Technology, Northwest A&F University, Yangling, China; 5 Hunan Provincial Key Laboratory for Germplasm Innovation and Utilization of Crop, RuanDa Road# 129, Changsha, Hunan, China; University of Auvergne, France

## Abstract

This study was conducted to investigate serum amino acids profile in dextran sulfate sodium (DSS)-induced colitis, and impacts of graded dose of arginine or glutamine supplementation on the colitis. Using DSS-induced colitis model, which is similar to human ulcerative colitis, we determined serum profile of amino acids at day 3, 7, 10 and 12 (5 days post DSS treatment). Meanwhile, effects of graded dose of arginine (0.4%, 0.8%, and 1.5%) or glutamine (0.5%, 1.0% and 2.0%) supplementation on clinical parameters, serum amino acids, colonic tight junction proteins, colonic anti-oxidative indicators [catalase, total superoxide dismutase (T-SOD), glutathione peroxidase (GSH-Px)], colonic pro-inflammatory cytokines [interleukin-1 beta (IL-1β), IL-6, IL-17 and tumor necrosis factor alpha (TNF-α)] in DSS-induced colitis were fully analyzed at day 7 and 12. Additionally, the activation of signal transduction pathways, including nuclear factor kappa B (NF-κB), mitogen-activated protein kinases (MAPK), phosphoinositide-3-kinases (PI3K)/PI3K-protein kinase B (Akt), and myosin light chain kinase (MLCK)- myosin light chain (MLC20), were analyzed using immunoblotting. Serum amino acids analysis showed that DSS treatment changed the serum contents of amino acids, such as Trp, Glu, and Gln (P<0.05). Dietary arginine or glutamine supplementation had significant (P<0.05) influence on the clinical and biochemical parameters (T-SOD, IL-17 and TNF-α) in colitis model. These results were associated with colonic NF-κB, PI3K-Akt and MLCK signaling pathways. In conclusion, arginine or glutamine could be a potential therapy for intestinal inflammatory diseases.

## Introduction

Inflammatory bowel diseases (IBD), including ulcerative colitis (UC) and Crohn's disease, are chronic gastrointestinal disorders characterized by chronic intestinal inflammation. The pathogenesis of IBD remains unclear, but imbalance between pro-inflammatory mediators, i.e. reactive oxygen mediators and cytokines, and anti-inflammatory responses is considered to be a key factor in the development and perpetuation of IBD [1]. Although the understanding of the pathogenesis in IBD is progressing, new therapeutic strategies continue to be investigated. Nutrients, capable of control the pro-inflammation, are shown to be beneficial in spontaneous and induced colitis models [2]. For example, vitamin D provides significantly beneficial role in IBD via mediating the expressions of target genes, including pro-inflammatory cytokines [3]–[5]. Besides that, compelling investigations have indicated that arginine or glutamine might be a good candidate for IBD treatment with low risk [6], [7]. For instance, post-treatment with alanyl-glutamine attenuates inflammatory response in dextran sulfate sodium (DSS)-induced colitis by suppressing Th17-associated cytokine expressions, reducing macrophage infiltration into the peritoneal cavity, and decreasing pro-inflammatory cytokine production in the colon [8]. Meanwhile, arginine supplementation improves the clinical parameters, including mortality, body weight and colon weight, and lowers the colonic permeability and the number of myeloperoxidase-positive neutrophils in colitis induced by DSS [9]. These well-designed investigations have indicated that these amino acids supplementation could be a potential therapy for IBD.

However, before recommending its use in clinical application, it is important to elucidate several important points. Firstly, it is necessary to analyze the serum profile of amino acids in the process of colonic injury and the recovery from IBD. Meanwhile, it is fruitful to investigate the suitable time, dose and means of arginine or glutamine supplementation to optimize its functions in IBD. Additionally, the biological mechanism of glutamine or arginine supplementation for IBD needs further elucidation. Thus, in the current study, we firstly determined the serum profile of amino acids in DSS-induced colitis in mice. Then, the impact of graded dose of arginine or glutamine supplementation on murine colitis caused by DSS was studied. The measured variables include serum amino acids, clinical parameters, antioxidative indicators [catalase, total superoxide dismutase (T-SOD), glutathione peroxidase (GSH-Px)], tight junction proteins [occludin, claudin-1, zonula occludens-1(ZO-1)], pro-inflammatory cytokines [interleukin-1 beta (IL-1β), IL-6, IL-17 and tumor necrosis factor alpha (TNF-α)] and signaling pathways [nuclear factor kappa B (NF-κB), mitogen-activated protein kinases (MAPK), phosphoinositide-3-kinases(PI3K)/PI3K-protein kinase B (Akt), myosin light chain kinase (MLCK)- myosin light chain (MLC20)].

## Materials and Methods

### Antibodies

Antibodies specific against occludin, claudin-1, zonula occludens-1 (ZO1), myosin light chain kinase (MLCK), myosin light chain (MLC20), p85, p-Akt, JNK and p-JNK were purchased from Santa Cruz Biotechnology, Inc.(Dallas, Texas, USA). Antibodies against ERK1/2, p-ERK1/2, p-38, p-p-38, p-65 were purchased from Cell Signaling Technology (Danvers, MA, USA).

### Experiment design

400 female ICR (Institute for Cancer Research) mice (6-week-old) were purchased from SLAC Laboratory Animal Central (Shanghai, China). The mice were housed in a pathogen-free mouse colony (temperature, 20–30°C; relative humidity, 45–60%; lighting cycle, 12 h/d) and had free access to food and drinking water. Animals were randomly divided into eight groups: 1) all mice were treated with distilled water and basal diet (NC) (n = 80); 2) all mice were treated with basal diet and distilled water containing 5% (wt/vol) DSS (MW 5000, KAYON Bio. Techology Co. Ltd) for 7 days (DS) (n = 80); 3) all mice were treated with DSS and dietary 0.5% glutamine (Ajinomoto Inc., Tokyo, Japan) supplementation (0.5-GS) (n = 40); 4) all mice were treated with DSS and dietary 1.0% glutamine supplementation (1.0-GS) (n = 40); 5) all mice were treated with DSS and dietary 2.0% glutamine supplementation (2.0-GS) (n = 40); 6) all mice were treated with DSS and dietary 0.4% arginine (Ajinomoto Inc., Tokyo, Japan) supplementation (0.4-AS) (n = 40); 7) all mice were treated with DSS and dietary 0.8% arginine supplementation (0.8- AS) (n = 40); 8) all mice were treated with DSS and dietary 1.5% arginine supplementation (1.5-AS) (n = 40). In arginine or glutamine supplementation groups, mice received arginine or glutamine from same day treating with DSS (day 1) to 5 days post DSS treatment (day 12). 20 mice from NC and DS groups were killed at day 3, 7, 10 and 12 to collect serum and colon for further analyses. Meanwhile, 20 mice in different dose of arginine or glutamine supplementation groups were killed at day 7 and 12 for serum and colon collection. Moreover, body weights were recorded daily during the experimental period, and the length and weight of colon were measured during the sample collection. After experiment, disease activity index (DAI) scores were analyzed to assess the severity of colitis. The scoring system was based on body weight gain, colon length, colon weight, and colonic inflammatory score via histological evaluation [10], [11]. This study was performed according to the guidelines of the Laboratory Animal Ethical Commission of the Chinese Academy of Sciences, and approved by Laboratory Animal Ethical Commission of the Institute of Subtropical Agriculture, Chinese Academy of Sciences (0207).

### Serum amino acid analysis

Before slaughter, six blood samples in each group at each time point were collected with orbital blooding. Then serum was separated with centrifugation at 3,500×g for 15 min at 4°C and stored at −20°C. Serum amino acids were analyzed with isotope dilution liquid chromatography-mass spectrometry methods by Beijing Amino Medical Research CO., LTD, Beijing, China.

### Real-time quantitative (RT-PCR)

Total RNA was isolated from liquid nitrogen frozen and ground colon with TRIZOL regent (Invitrogen, USA) and then treated with DNase I (Invitrogen, USA) according to the manufacturer's instructions. Synthesis of cDNA was performed with oligo (dT) 20 and Superscript II reverse transcriptase (Invitrogen, USA).

Primers (Table 1) were designed with Primer 5.0 according to mouse gene sequence. β-actin was used as an internal control to normalize target gene transcript levels. Real-time PCR was performed according to our previous study [12]. Briefly, 1 µl cDNA template was added to a total volume of 10 µl containing 5 µl SYBR Green mix, 0.2 µl Rox, 3 µl ddH2O, and 0.4 µl each of forward and reverse primers. We used the following protocol: (i) pre-denaturation program (10 s at 95°C); (ii) amplification and quantification program, repeated 40 cycles (5 s at 95°C, 20 s at 60°C); (iii) melting curve program (60–99°C with a heating rate of 0.1°C/s and fluorescence measurement). The relative expression was expressed as a ratio of the target gene to the control gene using the formula 2-(ΔΔCt), where ΔΔCt =  (CtTarget - Ctβ-actin)treatment - (CtTarget - Ctβ-actin)control. Relative expression was normalized and expressed as a ratio to the expression in the control group.

**Table 1:** Primers used in this study.

Gene	ID	Nucleotide sequence of primers (5′–3′)	Product Length	Ref.
β-actin	NM_007393.3	F:GTCCACCTTCCAGCAGATGT R:GAAAGGGTGTAAAACGCAGC	117	[38]
IL-1β	NM_008361.3	F:ATGAAAGACGGCACACCCAC R:GCTTGTGCTCTGCTTGTGAG	175	This study
IL-6	NM_031168.1	F:TGCAAGAGACTTCCATCCAGT R:GTGAAGTAGGGAAGGCCG	71	This study
IL-17	NM_010552.3	F:TACCTCAACCGTTCCACGTC R:TTTCCCTCCGCATTGACAC	119	This study
IL-18	NM_008360.1	F:AGACAACTTTGGCCGACTTC R:CCTTCACAGAGAGGGTCACA	203	This study
TNF-α	NM_013693.2	F:AGGCACTCCCCCAAAAGATR:TGAGGGTCTGGGCCATAGAA	192	This study

### Immunoblotting

Western blot analysis was conducted according to a previous study [13]. Briefly, the equal amounts of proteins obtained from cytoplasmic or nuclear fractions were separated by a reducing SDS-PAGE electrophoresis. The proteins were transferred onto PVDF membranes (Millipore, MA, USA) and blocked with 5% non-fat milk in Tris-Tween buffered saline buffer (20 mM Tris, pH 7.5,150 mM NaCl, and 0.1% Tween-20) for 3 hr. The primary antibodies were incubated overnight at 4°C; the HRP-conjugated secondary antibodies were subsequently incubated for 1 hr at room temperature before developing the blots using Alpha Imager 2200 software (Alpha Innotech Corporation, CA, USA). We digitally quantified the resultant signals and normalized the data to the proliferating cell nuclear antigen (PCNA) or actin abundance. PCNA or actin was used as an internal loading control for nuclear and cytoplasmic protein fractions, respectively.

### Catalase,T-SOD and GSH-Px activity analysis

The whole colon tissue was sampled from each mouse and homogenized (1 g/10 ml physiological saline solution) on ice. The homogenized solution centrifuged at 3,500×g for 15 min at 4°C, and the resultant liquid supernatant stored at −20°C. Catalase, total superoxide dismutase (T-SOD) activity and glutathione peroxidase (GSH-Px) in the colon were measured using spectrophotometric kits in accordance with the manufacturer's instructions (Nanjing Jiangcheng Biotechnology Institute, China)

### Morphological analyses

For light microscopic observation, the colon tissues were fixed with 10% formalin in PBS at 4°C, dehydrated in a graded series of ethanol, and then embedded in paraffin wax. The tissues were sectioned at 5 µm thick and mounted on slides. They were dewaxed, hydrated, and then stained with Hematoxylin-Eosin.

### Immunohistochemistry analyses

Tissues obtained from the colon of mice were fixed in 10% buffered formalin for 24 hr at room temperature and embedded in paraffin. The evaluations of occludin, ZO-1 and claudin-1 were made on 3 mm paraffin embedded slides, the sections were dewaxed in xylene, rehydrated in an ascendent ethanol scale and pre-treated in a microwave oven (two cycles for 5 minutes each at 780 W, in EDTA buffer, pH 8.0). Endogen biotin and non-specific signals were blocked with appropriated reagents. For immunohistochemistry, the treated slides were incubated with primary antibodies for 2 hr at room temperature in a humid chamber, washed in PBS, and visualized by biotinylated secondary antibodies followed by incubation with HRP-Conjugated Streptavidin for 30 min (R&D Systems, London, UK). The chromogen was 3, 39-diaminobenzidine free base (DAB).

### Statistical analysis

Data were expressed as means ± the standard error of the mean (SEM). All statistical analyses were performed using the SPSS 16.0 software (Chicago, IL, USA). Data were analyzed between NC and DS groups by the Student's *t*-test, while among NC, DS, AS and GS groups by the One-Way ANOVA method. Differences with P<0.05 were considered significant.

## Results

### Serum amino acids profile after DSS treatment

DSS induced colitis was used as a murine colonic injury and repair model for its similarities to human UC. The serum profile of amino acids was detected at day 3, 7, 10 and 12. At day 3, DSS significantly (P<0.05) decreased serum levels of Trp (tryptophane) and Aad (α-aminoadopic acid), while remarkably (P<0.05) promoted serum levels of bAib (β-aminoisobutyric acid), bAla (β-alanine) and Gln (glutamine), and had little effect on serum profile of other amino acids (Table 2 and Table S1). At day 7, DSS treatment significantly decreased (P<0.05) serum levels of Trp, GABA (γ-amino-n-butyric acid), 1MHis (1-methyl-L-histidine), Glu (glutamic acid) and Asp (Aspartic acid), while increased the serum level of bAla (beta-alanine), and had little effect on serum levels of other amino acids (Table 2 and Table S1). No significant difference was found about serum profile of amino acids between control and DS groups at day 10 and 12, except that serum level of Gln significantly (P<0.05) lowered in DS group at day 10 and 12 (Table 2 and Table S1). Meanwhile, arginine availability index (AAI) in DS group was significant (P<0.05) lower than that in NC group at day 12, while had no significant difference at day 3, 7 and 10 (Table 2).

**Table 2:** Serum amino acids profile in DSS treated group and control group.

	D3	D7	D10	D12
**Trp**				
Control	26.87±2.89a	19.69±1.32a	22.45±1.57	19.07±1.52
DS	13.93±0.95b	13.43±0.69b	19.06±2.15	14.72±1.15
**bAib**				
Control	0.18±0.01b	0.47±0.02	0.48±0.03	0.51±0.02b
DS	0.25±0.01a	0.50±0.01	0.45±0.01	0.67±0.02a
**GABA**				
Control	0.20±0.03	0.36±0.60a	0.09±0.01	0.14±0.03
DS	0.30±0.01	0.23±0.08b	0.12±0.03	0.17±0.04
**Aad**				
Control	1.18±0.28a	0.83±0.13	0.60±0.06	0.48±0.04
DS	0.63±0.04b	0.87±0.12	0.62±0.07	0.42±0.05
**1MHis**				
Control	0.86±0.09	1.06±0.03a	1.01±0.07	1.13±0.06
DS	0.87±0.03	0.81±0.06b	1.02±0.08	1.06±0.06
**Glu**				
Control	36.69±3.63	41.32±2.02a	35.37±2.71	43.27±3.55
DS	37.09±1.64	31.88±2.76b	37.06±2.87	36.40±1.43
**bAla**				
Control	1.03±0.04b	0.89±0.04b	0.76±0.08	0.72±0.02b
DS	1.354±0.06a	1.03±0.02a	0.65±0.04	0.89±0.04a
**Asp**				
Control	4.65±0.65	7.71±0.39a	4.85±0.44	6.97±0.63
DS	4.56±0.48	4.40±0.60b	4.60±0.43	7.58±0.76
**Gln**				
Control	60.95±4.06b	41.17±2.26	57.11±2.90a	62.50±6.87a
DS	82.68±3.86a	57.05±5.72	43.77±3.47b	45.53±4.53b
**PEtN**				
Control	1.58±0.15	1.32±0.19	1.98±0.13b	2.09±0.33
DS	1.98±0.10	0.80±0.09	2.89±0.18a	1.95±0.26

Arginine availability index (AAI)  = serum [L-Arg]/([L-Orn] + [L-Lys])

Control 0.42±0.04 0.36±0.02 0.42±0.03 0.49±0.03a

DS 0.40±0.02 0.38±0.02 0.47±0.05 0.35±0.04b

Mice were treated with DSS (DS) or normal drinking (Control). Data are presented as mean ± SEM, *n* = 6, with a-b used to indicate a statistically significant difference from control (*P*<0.05, Student's t- test).

### Average weight gain, colon length, and DAI scores after DSS treatment

**Figure 1:**
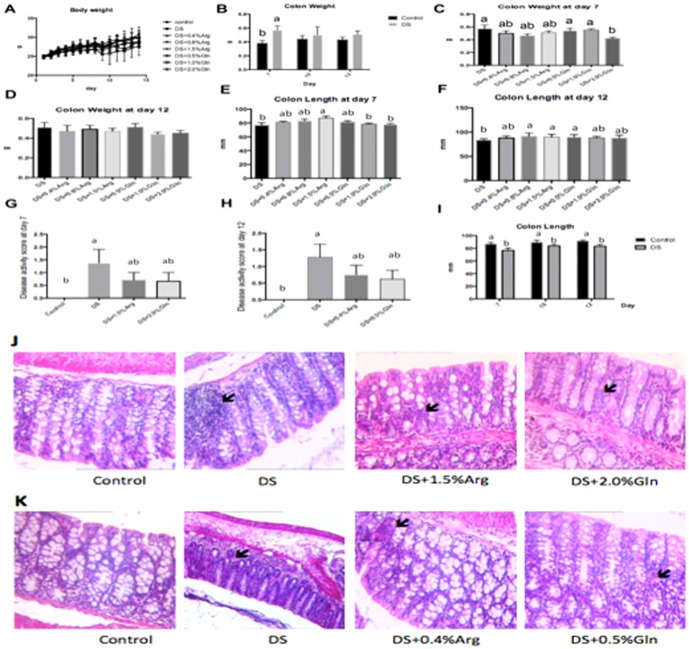
Arginine or glutamine supplementation affects the clinic parameters in dextran sulfate sodium colitis. A: Body weight was calculated in each group. B: Colon weight was measured in control and DS groups at day 7, 10 and 12. C: Colon weight was measured in each group at day 7. D: Colon weight was measured in each group at day 12. E: Colon length was collected in each group at day 7. F: Colon length was collected in each group at day 12. G: Disease activity score was calculated in each group at day 7. H: Disease activity score was calculated in each group at day 12. I: Colon length was measured in control and DS groups at day 7, 10 and 12. J: Colonic morphological analyses with Hematoxylin-Eosin staining in each group at day 7. K: Colonic morphological analyses with Hematoxylin-Eosin staining in each group at day 12. Mice were treated with normal drinking (control), or distilled water containing 5% (wt/vol) dextran sulfate sodium (DS), or dextran sulfate sodium water with different dose of arginine supplementation in diet (DS+ Arg), or dextran sulfate sodium water with different dose of glutamine supplementation in diet (DS+ Gln). Data are presented as mean ± SEM, *n* = 6, with a-c used to indicate a statistically significant difference (*P*<0.05, one way ANOVA method).

Previous study reported that DSS causes weight loss in mice [8]. Thus, this study also calculated the average weight gain (AWG) from day 1 to 12. No significant difference was found between two groups at this period (Figure 1A). However, the results indicated that DSS significantly (P<0.05) lowered the daily weight gain at day 7, compared to normal mice (−1.0±0.10 vs. −0.12±0.09). Meanwhile, DSS significantly (P<0.05) shortened colon length at day 7, 10 and 12 (Figure 1I), and increased colon weight at day 7 (Figure 1B), compared to those in NC group. Moreover, compared with NC group, there was a marked neutrophilic infiltration after exposure to DSS at day 7 and 12 (Figure 1 J and K). Based on these data, we analyzed the DAI score in colon after DSS treatment. As shown in figure 1G and H, mouse in DS group exhibited a higher (P<0.05) DAI score compared with that in control group at day 7 and 12, respectively.

### Average weight gain and colon length after amino acids supplementation

Firstly, we calculated the average weight gain and colonic parameters with graded dose of arginine or glutamine supplementation in mouse model with DSS-induced colitis to explore the effects of glutamine or arginine supplementation on IBD. About weight gain, no significant difference was found among graded dose of arginine or glutamine supplementation (Figure 1A). For colon weight, graded dose of arginine supplementation had little effect on colon weight at day 7 and 12 (Figure 1 C and D). However, dietary 2.0% glutamine supplementation significantly (P<0.05) decreased the colon weight at day 7, compared to that in DS group (Figure 1C). For colon length, at day 7, dietary 1.5% arginine supplementation significantly (P<0.05) increased the colon length, while other dose of arginine supplementation had little effect on colon length, compared to those at DS (Figure 1E). Glutamine supplementation had little effect on colon length compared to those in DS (Figure 1E). At day 12, colon length in 0.8-AS, or 1.5-AS, or 0.5-GS group was significant (P<0.05) longer than those in DS group (Figure 1F). Collectively, It seems that higher dose of glutamine (2.0%) or arginine (1.5%) supplementation is better than lower one in mouse model with DSS-induced colitis at first week, while lower dose of glutamine (0.5%) or arginine (0.4%) supplementation is appropriate at recover period from DSS-induced colitis. Indeed, HE staining also demonstrated that arginine or glutamine supplementation remarkably alleviated neutrophilic infiltration in the colon at day 7 (higher dose of supplementation, Figure 1J) and 12 (lower dose of supplementation, Figure 1K). However, arginine or glutamine supplementation failed to significantly decrease the DAI at day 7 and 12 (Figure 1J and K).

### Serum amino acids profile after amino acids supplementation

To monitor the effect of glutamine or arginine supplementation on serum profile of amino acids in mouse colitis induced by DSS, we determined the serum profile of amino acids in DS, 2.0-GS and 1.5-AS groups at day 7, and in DS, 0.5-GS, and 0.4-AS groups at day 12. As shown in table 3, arginine supplementation significantly (P<0.05) decreased serum levels of various amino acids, including Trp, Phe (phenylalanine), Ile (isoleucine), Val (valine), Met (methionine), Lys (lysine), Pro (proline), Arg, Cit (citrulline) and Thr (threonine), while remarkably (P<0.05) increased serum levels of Abu (α-amino-n-butyric acid), 3MHis (3-methyl-L-histidine), His (histidine) and Hyp (hydroxy-L-proline), compared to those in DS group at day 7. Meanwhile, dietary glutamine supplementation also significantly (P<0.05) decreased serum levels of various amino acids, compared with those in DS group (Table 3). These amino acids include Trp, Phe, Leu, Ile, Val, Lys, Orn (ornithine), Pro, Arg, 1MHis, Cit and Thr. At same time, glutamine supplementation obviously (P<0.05) enhanced serum levels of Ser (serine), Hyp, Gly, His, 3MHis and Abu (Table 3). At day 12, serum levels of several amino acids, including His, Gln, Hyp, Lys, Orn, Abu, Arg, Asn (asparagine) and 3MHis, were much higher (P<0.05) in 0.4-AS group than those in DS group (Table 3). Meanwhile, arginine supplementation also significantly (P<0.05) decreased serum levels of Trp, 1MHis, Asp and Thr, compared with those in DS group at day 12 (Table 3). Glutamine supplementation significantly (P<0.05) increased serum profiles of various serum amino acids, including Phe, Leu (leucine), Met, Lys, Orn, Abu, Arg, 3MHis, His, Gln. Gly and Hyp, compared with those in DS group at day 12 (Table 3). Besides that, glutamine supplementation also remarkably (P<0.05) decreased serum levels of Trp, Thr and Asp, compared with those in DS group at day 12 (Table 3). Furthermore, arginine or glutamine supplementation significantly (P<0.05) increased AAI at day 7 and 12, compared to the DS group (Table 3).

**Table 3:** Serum amino acids profile with arginine or glutamine supplementation in DSS-induced colitis model.

		D7		D12
**Trp**				
DS		13.43±0.69a		14.72±1.15a
DS+Arg		6.87±0.50b		8.22±0.57b
DS+Gln		7.11±0.72b		8.10±0.44b
**Phe**				
DS		17.50±1.04a		14.05±0.62b
DS+Arg		12.62±0.42b		16.20±1.61ab
DS+Gln		11.61±1.10b		17.78±0.97a
**Leu**				
DS		30.46±2.27a		20.57±1.52b
DS+Arg		25.60±1.66ab		27.73±3.36ab
DS+Gln		22.72±2.99b		34.28±1.51a
**Ile**				
DS		19.43±1.48a		13.14±0.75
DS+Arg		13.24±1.33b		15.87±2.43
DS+Gln		10.72±1.28b		16.33±1.09
**Tyr**				
DS		20.53±1.99		22.09±1.71
DS+Arg		19.63±1.73		21.72±3.20
DS+Gln		21.12±2.29		26.17±1.88
**Val**				
DS		44.78±3.72a		29.37±2.32
DS+Arg		24.14±0.62b		33.03±5.69
DS+Gln		21.20±2.82b		35.04±2.56
**Met**				
DS		40.73±4.48a		23.73±2.93b
DS+Arg		23.36±1.02b		40.12±9.03ab
DS+Gln		30.05±5.96ab		41.02±5.32a
**Lys**				
DS		113.72±6.40a		88.66±5.44a
DS+Arg		44.47±2.82b		55.11±7.68b
DS+Gln		44.27±5.33b		65.17±5.37b
**Orn**				
DS		27.08±1.96ab		20.01±1.95b
DS+Arg		32.95±3.42a		29.16±5.41a
DS+Gln		24.56±2.96b		31.77±2.92a
**Pro**				
DS		25.99±1.80a		21.33±1.43
DS+Arg		16.45±1.55b		24.98±5.41
DS+Gln		18.68±1.88b		21.63±1.17
**Abu**				
DS		0.61±0.42b		0.63±0.06b
DS+Arg		3.95±1.05a		3.52±0.43a
DS+Gln		4.94±1.47a		2.97±0.43a
**Arg**				
DS		54.62±4.52a		43.32±2.93b
DS+Arg		39.16±2.38b		47.89±2.84b
DS+Gln		35.52±2.46b		62.09±5.76a
**3MHis**				
DS		0.52±0.04b		0.44±0.03b
DS+Arg		0.74±0.08a		0.74±0.05a
DS+Gln		0.87±0.07a		0.94±0.06a
**1MHis**				
DS		0.81±0.06a		1.06±0.06a
DS+Arg		0.76±0.06ab		0.85±0.05b
DS+Gln		0.57±0.07b		1.01±0.06ab
**Glu**				
DS		31.88±2.76		36.40±1.43
DS+Arg		27.03±1.60		32.57±2.10
DS+Gln		29.61±2.89		34.16±1.92
**Ala**				
DS		54.03±3.17		48.17±1.96
DS+Arg		54.54±4.89		54.25±8.67
DS+Gln		60.49±5.60		67.23±4.90
**Cit**				
DS		28.83±1.96a		21.28±0.67
DS+Arg		17.53±1.19b		19.88±2.32
DS+Gln		17.42±2.04b		23.79±2.94
**Thr**				
DS		54.67±2.88a		36.29±3.23a
DS+Arg		20.51±1.35b		25.96±3.97b
DS+Gln		23.82±4.56b		25.30±3.88b
**His**				
DS		23.86±2.02c		18.10±1.98b
DS+Arg		51.67±3.89a		49.21±4.73a
DS+Gln		40.60±6.49b		50.07±5.28a
**Asp**				
DS		4.40±0.60		7.58±0.76a
DS+Arg		3.43±0.34		4.61±0.65b
DS+Gln		3.66±0.69		4.60±0.49b
**Gln**				
DS		57.05±5.72		45.53±4.53b
DS+Arg		65.86±5.03		87.52±8.27a
DS+Gln		61.03±3.26		88.48±11.77a
**Gly**				
DS		33.63±1.57b		28.93±1.05b
DS+Arg		31.21±2.06b		27.99±4.05b
DS+Gln		58.13±3.72a		36.77±3.75a
**Hyp**				
DS		3.96±0.31b		3.31±0.15b
DS+Arg		12.88±0.98a		12.24±1.29a
DS+Gln		13.94±1.22a		12.52±0.96a
**Ser**				
DS		28.67±1.15b		25.86±1.88
DS+Arg		30.57±3.67b		30.80±3.36
DS+Gln		39.32±3.21a		30.24±3.36
**Asn**				
DS		9.63±0.76		7.24±0.30b
DS+Arg		8.84±0.65		10.20±1.03a
DS+Gln		9.32±1.04		9.18±0.98ab

Arginine availability index (AAI)  = serum [L-Arg]/([L-Orn] + [L-Lys])

DS 0.38±0.02b 0.35±0.04b

DS+Arg 0.52±0.03a 0.64±0.06a

DS+Gln 0.57±0.05a 0.59±0.03a

At day 7, serum amino acids profile in mice treated with DSS feeding basal diet (DS), or 1.5% arginine supplementation (DS+Arg), or 2.0% arginine supplementation (DS+Gln), were detected. At day 12, serum amino acids profile in mice treated with DSS feeding basal diet (DS), or 0.4% arginine supplementation (DS+Arg), or 0.5% glutamine supplementation at day 12 (DS+Gln), were detected. Data are presented as mean ±SEM, *n* = 6, with a-c used to indicate a statistically significant difference (*P*<0.05, one way ANOVA method).

### Catalase, T-SOD and GSH-Px activity

**Figure 2:**
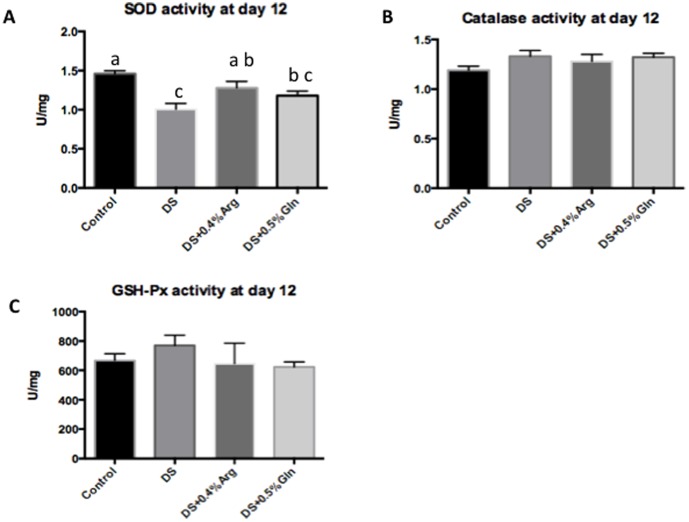
Antioxidant enzymes activities in each group. A: SOD activity in each group. B: Catalase activity in each group. C: GSH-Px activity in each group. Mice were treated with normal drinking (control), or distilled water containing 5% (wt/vol) dextran sulfate sodium (DS), or dextran sulfate sodium water with dietary 0.4% arginine supplementation (DSS+0.4% Arg), or dextran sulfate sodium water with dietary 0.5% glutamine supplementation (DS+ 0.5% Gln). Data are presented as mean ± SEM, *n* = 6, with a-c used to indicate a statistically significant difference (*P*<0.05, one way ANOVA method). T-SOD: total superoxide dismutase, GSH-Px: glutathione peroxidase.

Oxidative stress has been involved in the pathogenesis of inflammatory bowel disease, and investigations have found that the inflamed mucosa increases the production of reactive oxygen metabolities, such as superoxide, hydroxyl radical and hydrogen peroxide [14]. Thus, we detected some factors in anti-oxidative system, including catalase, T-SOD and GSH-Px. No significant difference was found between NC and DS group, or between DS and 0.4-AS or 0.5-GS groups about catalase (Figure 2 B) and GSH-Px (Figure 2 C) at day 12. However, DSS treatment significantly (P<0.05) decreased T-SOD activity in the colon, compared to those in NC group (Figure 2 A). Arginine supplementation significantly (P<0.05) reversed the decrease of T-SOD caused by DSS, while glutamine supplementation had little effect on T-SOD activity, compared to those in DS group (Figure 2 A).

### Pro-inflammatory cytokines in colon

An increased production of proinflammatory cytokines [TNF-α, IL-1β, IL-6, IL-17] has been reported in patients with IBD. In this study, we found that arginine supplementation significantly (P<0.05) promoted the colonic expressions of IL-17 and TNF-α at day 7, compared to those in DS group (Table 4). At day 12, arginine supplementation significantly (P<0.05) reduced the expressions of IL-17 and TNF-α in the colon (Table 4). Also, glutamine supplementation remarkably (P<0.05) promoted the colonic expressions of TNF-α, IL-1β and IL-17 at day 7, and expression of TNF-α at day 12, compared to the DS group (Table 4).

**Table 4:** Pro-inflammatory cytokines expression with arginine or glutamine supplementation in DSS-induced colitis model.

	IL-1β	IL-6	IL-17	TNF-α
**D 7**				
DS	1.00±0.02b	1.00±0.02	1.00±0.03c	1.00±0.02b
DS+Arg	1.06±0.02b	1.00±0.02	1.12±0.02b	1.35±0.03a
DS+Gln	1.14±0.03a	1.01±0.02	1.24±0.02a	1.27±0.06a
**D 12**				
DS	1.00±0.01	1.00±0.01	1.00±0.01a	1.00±0.03b
DS+Arg	1.02±0.02	1.02±0.03	0.86±0.02b	0.86±0.02c
DS+Gln	1.00±0.02	1.01±0.02	1.20±0.02a	1.18±0.03a

At day 7, colonic proinflammatory cytokines production in mice treated with DSS feeding basal diet (DS), or 1.5% arginine supplementation (DS+Arg), or 2.0% arginine supplementation (DS+Gln), were detected. At day 12, colonic proinflammatory cytokines production in mice treated with DSS feeding basal diet (DS), or 0.4% arginine supplementation (DS+Arg), or 0.5% glutamine supplementation at day 12 (DS+Gln), were detected. Data are presented as mean ±SEM, *n* = 6, with a-c used to indicate a statistically significant difference (*P*<0.05, one way ANOVA method). IL: Interleukin, TNF: tumor necrosis factor.

### MAPK and NF-κB pathway

**Figure 3:**
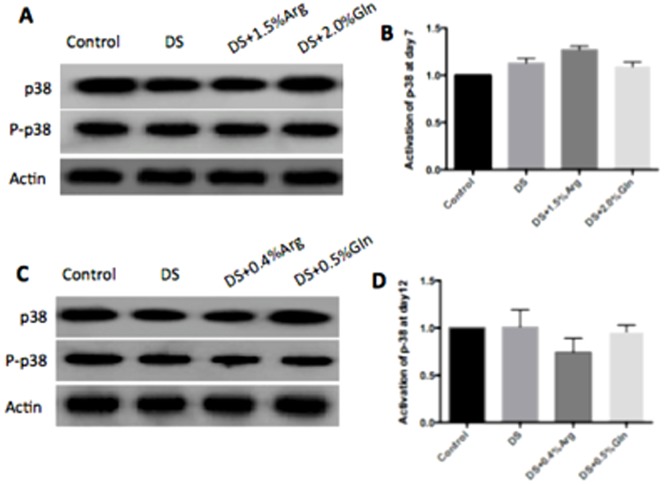
Activation of p38 in colon. A: Immunoblotting of total and phosphorylated p38 at day 7. B: The ratio of phosphorylated p38 to total p38 from data shown in A. C: Immunoblotting of total and phosphorylated p38 at day 12. D: The ratio of phosphorylated p38 to total p38 from data shown in C. Mice were treated with normal drinking (control), or distilled water containing dextran sulfate sodium (DS), or dextran sulfate sodium water with dietary 0.4% arginine supplementation (DSS+0.4% Arg), or dextran sulfate sodium water with dietary 1.5% arginine supplementation (DSS+1.5% Arg), or dextran sulfate sodium water with dietary 0.5% glutamine supplementation (DS+ 0.5% Gln), or dextran sulfate sodium water with dietary 2.0% glutamine supplementation (DS+ 2.0% Gln). Data are presented as mean ±SEM, *n* = 5.

**Figure 4:**
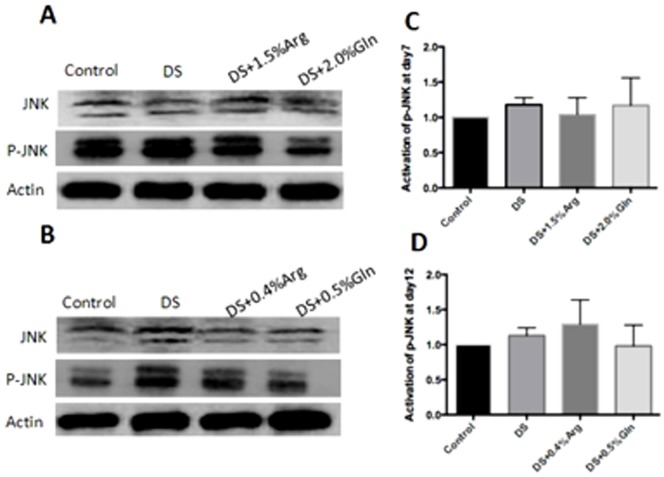
Activation of JNK in colon. A: Immunoblotting of total and phosphorylated JNK at day 7. B: Immunoblotting of total and phosphorylated JNK at day 12. C: The ratio of phosphorylated JNK to total JNK from data shown in A. D: The ratio of phosphorylated JNK to total JNK from data shown in B. Mice were treated with normal drinking (control), or distilled water containing dextran sulfate sodium (DS), or dextran sulfate sodium water with dietary 0.4% arginine supplementation (DSS+0.4% Arg), or dextran sulfate sodium water with dietary 1.5% arginine supplementation (DSS+1.5% Arg), or dextran sulfate sodium water with dietary 0.5% glutamine supplementation (DS+ 0.5% Gln), or dextran sulfate sodium water with dietary 2.0% glutamine supplementation (DS+ 2.0% Gln). Data are presented as mean ±SEM, *n* = 5. JNK: c-Jun N-terminal kinase.

**Figure 5:**
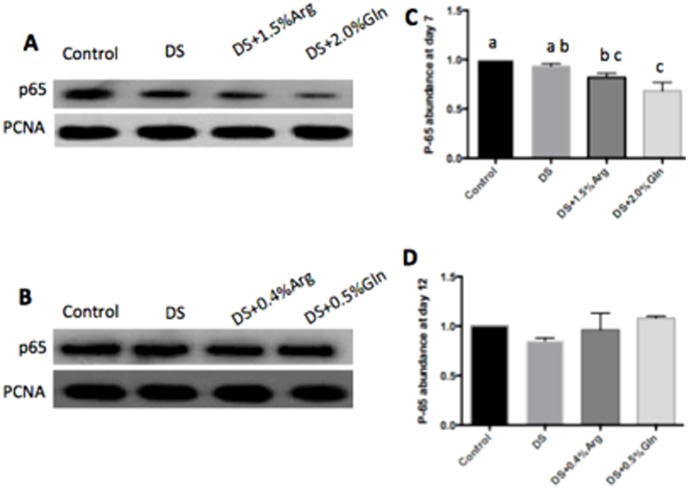
Abundance of p65 in the colon. A: Immunoblotting of colonic nuclear p65 at day 7. B: Immunoblotting of colonic nuclear p65 at day 12. C: Quantification of relative colonic nuclear p65 abundance from data shown in A. D: Quantification of relative colonic nuclear p65 abundance from data shown in B. Mice were treated with normal drinking (control), or distilled water containing dextran sulfate sodium (DS), or dextran sulfate sodium water with dietary 0.4% arginine supplementation (DSS+0.4% Arg), or dextran sulfate sodium water with dietary 1.5% arginine supplementation (DSS+1.5% Arg), or dextran sulfate sodium water with dietary 0.5% glutamine supplementation (DS+ 0.5% Gln), or dextran sulfate sodium water with dietary 2.0% glutamine supplementation (DS+ 2.0% Gln). Data are presented as mean ±SEM, *n* = 5, with a-b used to indicate a statistically significant difference (*P*<0.05, one way ANOVA method). PCNA: proliferating cell nuclear antigen.

The activation of MAPK and NF-κB pathways has been implicated in the pathogenesis of IBD [15]. Dietary arginine or glutamine supplementation had little effect on the activation of p38 and JNK pathway in the colon at day 7 and 12 (Figure 3 and 4). About NF-κB pathway, dietary glutamine supplementation significantly (P<0.05) inhibited the activation of colonic nuclear p65 at day 7, while had little effect on p65 abundance at day 12, compared to DS group (Figure 5). Arginine supplementation had little effect on the colonic nuclear p65 abundance at day 7 and 12, compared to DS group (Figure 5).

### PI3K-Akt and MLCK-MLC20 pathway

**Figure 6:**
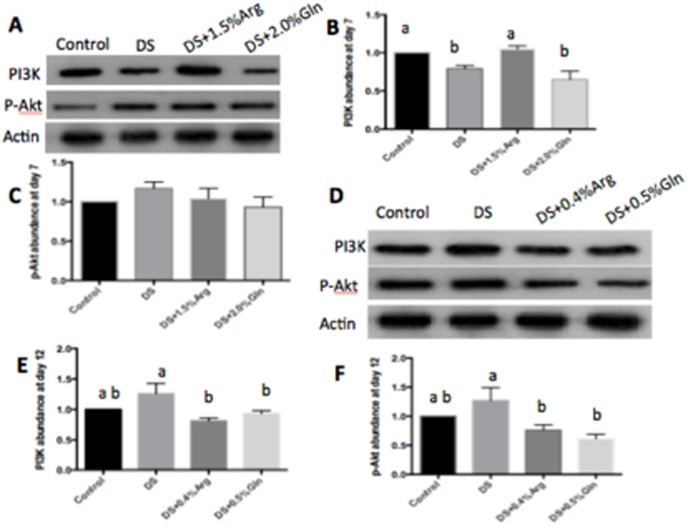
Activation of colonic PI3K-Akt pathway. A: Immunoblotting of colonic PI3K and p-Akt at day 7. B: Quantification of relative PI3K abundance from data shown in A. C: Quantification of relative P-Akt abundance from data shown in A. D: Immunoblotting of colonic PI3K and p-Akt at day 12. E: Quantification of relative PI3K abundance from data shown in D. F: Quantification of relative P-Akt abundance from data shown in E. Mice were treated with normal drinking (control), or distilled water containing dextran sulfate sodium (DS), or dextran sulfate sodium water with dietary 0.4% arginine supplementation (DSS+0.4% Arg), or dextran sulfate sodium water with dietary 1.5% arginine supplementation (DSS+1.5% Arg), or dextran sulfate sodium water with dietary 0.5% glutamine supplementation (DS+ 0.5% Gln), or dextran sulfate sodium water with dietary 2.0% glutamine supplementation (DS+ 2.0% Gln). Data are presented as mean ±SEM, *n* = 5, with a-b used to indicate a statistically significant difference (*P*<0.05, one way ANOVA method). PI3K: Phosphatidylinositide 3-kinases, Akt: protein kinases B.

PI3K-Akt pathway may be an important factor in maintenance of inflammation and tissue damage in IBD because it is responsible for the migration of leukocytes from the bloodstream to sites of injury or infection [16]. At day 7, arginine supplementation significantly (P<0.05) increased the abundance of colonic PI3K, while glutamine supplementation had little effect on colonic PI3K-Akt pathway, compared to those in DS group (Figure 6). However, dietary arginine or glutamine supplementation significantly (P<0.05) inhibited the activation of colonic PI3K-Akt pathway at day 12, compared to the DS group (Figure 6).

**Figure 7:**
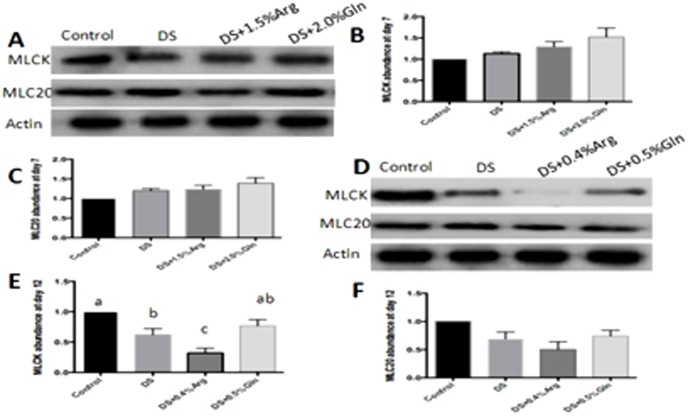
Activation of colonic MLCK-MLC20 pathway. A: Immunoblotting of colonic MLCK-MLC20 at day 7. B: Quantification of relative MLCK abundance from data shown in A. C: Quantification of relative MLC20 abundance from data shown in A. D: Immunoblotting of colonic MLCK and MLC20 at day 12. E: Quantification of relative MLCK abundance from data shown in D. F: Quantification of relative MLC20 abundance from data shown in E. Mice were treated with normal drinking (control), or distilled water containing dextran sulfate sodium (DS), or dextran sulfate sodium water with dietary 0.4% arginine supplementation (DSS+0.4% Arg), or dextran sulfate sodium water with dietary 1.5% arginine supplementation (DSS+1.5% Arg), or dextran sulfate sodium water with dietary 0.5% glutamine supplementation (DS+ 0.5% Gln), or dextran sulfate sodium water with dietary 2.0% glutamine supplementation (DS+ 2.0% Gln). Data are presented as mean ±SEM, *n* = 5, with a-c used to indicate a statistically significant difference (*P*<0.05, one way ANOVA method). MLCK: Myosin light-chain kinase. MLC20: Myosin light chain 20.

MLCK-MLC20 pathway also plays an important role on IBD because this pathway involves in tight junction dysregulation and epithelial damage, resulting in barrier loss in patients with IBD. Glutamine supplementation had little effect on this pathway at colon at day 7 and 12, compared to the DS group (Figure 7). Arginine supplementation significantly (P<0.05) decreased colonic MLCK abundance at day 12, while it had little effect on this pathway at day 7, compared with those in DS group (Figure 7).

### Expression of tight junction

**Figure 8:**
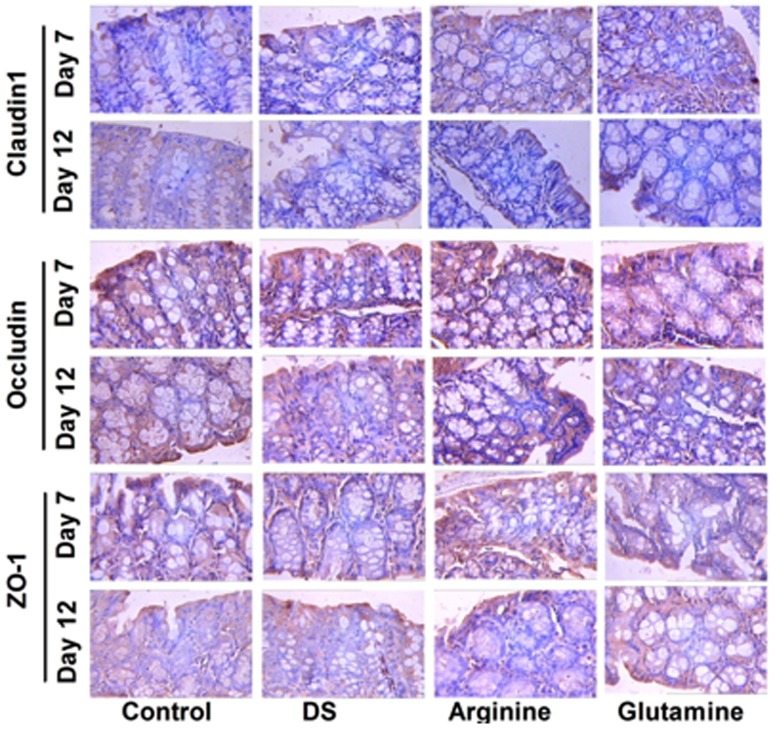
Tight junctions expression in the colon. Claudin1, occludin, and ZO-1 were analyzed with immunohistochemistry analyses in DSS-induced colitis model at day 7 and 12. Mice were treated with normal drinking (control), or distilled water containing dextran sulfate sodium (DS), or dextran sulfate sodium water with dietary arginine supplementation (Arginine), or dextran sulfate sodium water with dietary glutamine supplementation (Glutamine). The dosage of arginine supplementation at day 7 is 1.5%, while it is 0.4% at day 12. The dosage of glutamine supplementation at day 7 is 2.0%, while it is 0.5% at day 12. ZO-1: zonula occluden-1.

Tight junctions, the first physical barrier against a variety of pathogens, act as a main mechanism to maintain the homeostasis in gastrointestinal tract. We, hence, quantified the expressions of tight junctions (claudin1, occludin, and ZO1) via immunohistochemistry analyses. As shown in Figure 8, the expression of claudin-1 exhibited little difference between the control and DS group, while dietary supplementation with arginine and glutamine obviously enhanced its abundance at day 7 (Figure 8). At day 12, the expression of claudin-1 significantly decreased at DS group and amino acid groups compared with that at control group (Figure 8). However, we failed to notice a similar trend in the expressions of occludin (Figure 8) and ZO-1 (Figure 8).

## Discussion

Numerous compelling investigations have indicated that a metabolic alteration will occur in patients with IBD, resulting in the change of serum profile of amino acids. For example, one well-designed study has found that several serum amino acids, such as isoleucine and lysine, show a strong increase in patients with UC [17]. Similarly, another investigation has showed that plasma His concentrations in both CD and UC patients are significantly lower than the health patients [18]. Also, serum concentrations of L-Arg, L-Lys, L-Orn, L-Pro, and L-Cit increase in the mice treated with DSS, compared to untreated control mice [9]. However, in contrast to previous reports, we found that serum levels of Trp, Glu, Gln and AAI decrease in the mice treated with DSS. Besides that, no much difference is found between DSS treated and control mice. The rationale for this discrepancy remains unknown; perhaps it is related to different samples detected or the sensitivity of diverse detection methods. Indeed, similar to our discovery, other study has suggested that plasma concentration of Trp significantly decreases in IBD patients, and this decrease inversely correlates with disease activity [18]. Similarly, another study has also indicated that levels of glutamic acid and glutamine significantly decrease in the acute phase of colonic inflammation [19]. The underling biological mechanism for the decrease of Trp, Glu, Gln levels and AAI in DSS-induced colitis merits further investigation. To our knowledge, this is the first time to determine serum profile of amino acids in the progress of colitis and its recover in the mice explored to DSS. Our discovery may provide metabolic evidence for the beneficial functions of Arg, Glu and Gln supplementation in IBD patients. Furthermore, amino acids profiles in tissue or urine still need further investigate in the progress of IBD and its recovery.

Previous studies have indicated that DSS induces acute colitis and increases DAI score in ICR mice [20]–[22], we also found DSS treatment causes significant colonic damages to mice. Dietary arginine or glutamine supplementation obviously alleviates the damages caused by DSS. Indeed, increasing studies have demonstrated that arginine or glutamine supplementation is beneficial for decrease the weight loss or colonic damages in mice caused by DSS [9], [23], [24]. Interestingly, the beneficial function of arginine or glutamine supplementation in IBD depends on the supplemental dosage, which also differs from different period of the disease. Similarly, our previous study has indicated that the effect of glutamine supplementation on the burden of *Pasteurella multocida* and the expressions of its major virulence factors in mice is also dose and tissue dependent [25]. The exact mechanism may relate to the metabolism and balance of amino acids. To our knowledge, this is first study to investigate the effect of graded dose of arginine or glutamine supplementation on the pathogenesis of IBD. However, the optimal supplementation time for arginine or glutamine in IBD still needs further investigation. Metabolically, arginine or glutamine supplementation causes remarkably change of serum profile of amino acids at day 7 and 12. The explanation for this change is that arginine or glutamine supplementation will change carbohydrate metabolism, amino acid metabolism, and lipid metabolism, leading to the change of serum profile of amino acids [26]–[28]. Intriguingly, dietary arginine or glutamine supplementation significantly increases serum Gln content and AAI, which are lowered in our DSS-induced colitis model. This might be one of possibility that arginine or glutamine supplementation alleviates the colonic damage in mice caused by DSS. However, the metabolic profile in tissue and urine after arginine or glutamine supplementation merits further exploration in IBD.

Similar to previous studies [14], [29], DSS decreases colonic T-SOD activity in mice. Arginine supplementation reverses this decline at day 12 in DSS-induced colitis model, which may explain the beneficial role of arginine supplementation on colonic oxidative injury caused by DSS in mice. Indeed, our previous study also showed that arginine supplementation increases serum T-SOD activity in porcine circovirus infected pregnant mouse [30] and pig [31]. However, arginine or glutamine supplementation has little effect on colonic CAT and GHS-Px activities in DSS treated mice, thus it is interesting to explore the effect of arginine or glutamine supplementation on colonic reactive oxygen metabolities, such as superoxide, hydroxyl radical and hydrogen peroxide, because these substances are higher in IBD model than its control [14], [32].

Intriguingly, arginine or glutamine supplementation increases the production of colonic inflammatory cytokines, including IL-1β, IL-17, and TNF-α at day 7. This observation is much difference from the previous reports that arginine or glutamine supplementation significantly lowers these cytokines production [8], [9], [23]. The explanation for this difference remains unknown; perhaps it is related to the high dose of arginine (1.5% in diet) or glutamine (2.0% in diet) supplementation. In our other study, we also observed that higher (2.0%) dose of glutamine supplementation significantly promotes the pro-inflammation expression, compared to the lower dose of glutamine supplementation in the spleen in *Pasteurella multocida* infected mice[25]. Indeed, 0.4% arginine supplementation lowered the expressions of IL-17 and TNF-α, compared to the DS group at day 12. These results indicated that we should play more attention on the dose of arginine or glutamine supplementation. However, it remains to explore the effect of arginine or glutamine supplementation the production of anti-inflammatory cytokines (IL-4, IL-10). Another possibility is that DSS contamination on colonic tissues potently inhibits the RT-PCR amplification [33].

Glutamine supplementation inhibits the activation of NF-κB at day 7. Pervious compelling study also showed that glutamine significantly suppresses p65 protein expression in the colon when compared to that of colitis mice without treatment by glutamine [34]. However, it is interesting to explore the effect of glutamine supplementation on other components of NF-κB pathway in DSS-induced colitis model because glutamine could counteract activation of this pathway at multiple levels[7]. At day 12, arginine or glutamine supplementation could inactivate the colonic PI3K-Akt pathway, which adds more evidence to previous study [35] [36]. However, in DSS-induced colitis model, it will be fruitful to understand the effect of arginine or glutamine supplementation on other colonic signaling pathways, such as signal transducer and activator of transcription (STAT), activating protein-1 (AP-1), and peroxisome proliferator-activated receptor-γ (PPARγ), which play central role in intestinal inflammation [7]. Interestingly, arginine supplementation inhibits the activation of MLCK. MLCK is involved in cytokine-mediated regulation of the tight junction structure, resulting in reorganization of tight junction proteins [37]. In our study, we also found that arginine increases the expression of claudin-1 at day 7 after DSS treatment. Thus, it remains to know the effect of arginine supplementation in tight junctions function in DSS-induced colitis model.

In conclusion, the present study provides in *vivo* evidence that serum amino acids profile changes in DSS-induced colitis model. Meanwhile, arginine or glutamine supplementation can improve clinical and biochemical parameters, involving in NF-κB, PI3K-Akt and MLCK signaling pathways, in a murine colitis model of injury and repair with similarities to human UC. Moreover, the beneficial role of arginine or glutamine on DSS-induced colitis model depends on the supplemental dosage. The substantial effect of arginine or glutamine on intestinal inflammatory signaling pathways suggests the potential for broad applicability in other diseases associated with inflammation.

## Supporting Information

Table S1 **Serum amino acids profile in DSS treated group and control group.**
(DOCX)
