# Supplementary material for: Serum Amino Acids Profile and the Beneficial Effects of L-Arginine or L-Glutamine Supplementation in Dextran Sulfate Sodium Colitis
Source: PLoS One. 2014 Feb 5;9(2):e88335. doi: 10.1371/journal.pone.0088335 (PMC3914992; doi:10.1371/journal.pone.0088335)
Supplement: Table S1 — Serum amino acids profile in DSS treated group and control group. (DOCX) [file pone.0088335.s001.docx]

Table S1 Serum amino acids profile in DSS treated group and control group.

|  | **D3** | **D7** | **D10** | **D12** |
| --- | --- | --- | --- | --- |
| **Phe** |  |  |  |  |
| Control | 20.36±1.53 | 18.10±1.17 | 19.24±1.21 | 16.64±1.21 |
| DS | 20.55±1.56 | 17.50±1.04 | 20.64±1.77 | 14.05±0.62 |
| **Leu** |  |  |  |  |
| Control | 33.87±3.27 | 30.77±2.58 | 32.14±2.52 | 24.74±1.87 |
| DS | 34.26±3.60 | 30.46±2.27 | 30.42±1.74 | 20.57±1.52 |
| **Ile** |  |  |  |  |
| Control | 20.03±2.03 | 18.81±1.76 | 19.14±1.53 | 14.34±0.94 |
| DS | 20.63±2.35 | 19.43±1.48 | 18.67±1.21 | 13.14±0.75 |
| **Tyr** |  |  |  |  |
| Control | 26.61±1.55 | 20.09±1.96 | 20.01±1.37 | 17.71±1.84 |
| DS | 24.65±2.51 | 20.53±1.99 | 22.13±1.57 | 22.09±1.71 |
| **Val** |  |  |  |  |
| Control | 50.12±4.76 | 43.31±4.89 | 44.91±3.56 | 34.24±2.37 |
| DS | 48.83±5.52 | 44.78±3.72 | 43.81±2.66 | 29.37±2.32 |
| **Met** |  |  |  |  |
| Control | 59.34±6.69 | 46.38±7.07 | 45.18±5.24 | 33.06±6.08 |
| DS | 50.45±1.83 | 40.73±4.48 | 47.44±5.35 | 23.73±2.93 |
| **Lys** |  |  |  |  |
| Control | 125.70±6.37 | 113.63±12.52 | 112.43±10.23 | 94.35±8.87 |
| DS | 125.42±7.25 | 113.72±6.40 | 93.17±8.31 | 88.66±5.44 |
| **Cth** |  |  |  |  |
| Control | 0.69±0.06 | 0.44±0.04 | 0.43±0.07 | 0.45±0.03 |
| DS | 0.69±0.06 | 0.57±0.05 | 0.42±0.06 | 0.49±0.07 |
| **Orn** |  |  |  |  |
| Control | 26.11±2.18 | 23.78±3.91 | 23.89±2.39 | 18.80±2.24 |
| DS | 26.27±2.02 | 27.08±1.96 | 23.84±2.88 | 20.01±1.95 |
| **Pro** |  |  |  |  |
| Control | 32.24±2.15 | 27.63±2.92 | 25.21±1.65 | 19.97±2.04 |
| DS | 28.78±1.74 | 25.99±1.80 | 25.68±2.40 | 21.33±1.43 |
| **Hyl** |  |  |  |  |
| Control | 0.43±0.04 | 0.49±0.03 | 0.34±0.03 | 0.30±0.04 |
| DS | 0.44±0.05 | 0.52±0.05 | 0.39±0.04 | 0.31±0.01 |
| **Abu** |  |  |  |  |
| Control | 0.81±0.10 | 0.57±0.03 | 0.67±0.05 | 0.70±0.05 |
| DS | 0.67±0.02 | 0.61±0.42 | 0.63±0.04 | 0.63±0.06 |
| **Arg** |  |  |  |  |
| Control | 58.56±7.96 | 53.57±5.44 | 56.75±3.79 | 51.05±1.52 |
| DS | 60.49±4.45 | 54.62±4.52 | 53.91±3.28 | 43.32±2.93 |
| **Hcit** |  |  |  |  |
| Control | 0.17±0.03 | 0.14±0.02 | 0.14±0.01 | 0.12±0.01 |
| DS | 0.15±0.01 | 0.12±0.01 | 0.12±0.00 | 0.12±0.03 |
| **Ans** |  |  |  |  |
| Control | 0.19±0.03 | 0.14±0.01 | 0.14±0.02 | 0.09±0.01 |
| DS | 0.22±0.02 | 0.16±0.02 | 0.14±0.01 | 0.10±0.01 |
| **Car** |  |  |  |  |
| Control | 0.18±0.02 | 0.21±0.05 | 0.21±0.02 | 0.37±0.04 |
| DS | 0.19±0.01 | 0.20±0.03 | 0.18±0.01 | 0.31±0.02 |
| **3MHis** |  |  |  |  |
| Control | 0.52±0.02 | 0.53±0.03 | 0.47±0.06 | 0.40±0.02 |
| DS | 0.56±0.02 | 0.52±0.04 | 0.46±0.03 | 0.44±0.03 |
| **Ala** |  |  |  |  |
| Control | 66.85±3.20 | 58.14±4.66 | 58.97±4.32 | 50.78±5.84 |
| DS | 63.23±2.75 | 54.03±3.17 | 52.92±3.22 | 48.17±1.96 |
| **Cit** |  |  |  |  |
| Control | 32.92±2.43 | 26.77±2.70 | 28.94±1.86 | 21.55±1.42 |
| DS | 28.53±2.29 | 28.83±1.96 | 27.81±2.43 | 21.28±0.67 |
| **Thr** |  |  |  |  |
| Control | 53.50±4.03 | 51.66±6.03 | 53.50±3.70 | 44.64±4.58 |
| DS | 53.65±3.20 | 54.67±2.88 | 48.75±3.26 | 36.29±3.23 |
| **His** |  |  |  |  |
| Control | 26.56±1.33 | 22.80±1.28 | 26.87±1.79 | 23.32±2.27 |
| DS | 30.68±2.02 | 23.86±2.02 | 23.95±1.23 | 18.10±1.98 |
| **Gly** |  |  |  |  |
| Control | 32.65±1.87 | 33.19±2.63 | 30.36±1.66 | 27.88±2.46 |
| DS | 36.37±1.73 | 33.63±1.57 | 31.39±2.68 | 28.93±1.05 |
| **Hyp** |  |  |  |  |
| Control | 5.04±0.35 | 4.59±0.3846 | 3.4217±0.317 | 2.72±0.25516 |
| DS | 4.48±0.27 | 3.96±0.31 | 3.55±0.19 | 3.31±0.15 |
| **Ser** |  |  |  |  |
| Control | 39.55±3.15 | 31.40±2.34 | 31.56±1.72 | 32.02±3.65 |
| DS | 36.65±1.76 | 28.67±1.15 | 33.25±2.38 | 25.86±1.88 |
| **Asn** |  |  |  |  |
| Control | 11.57±0.55 | 9.70±0.71 | 9.40±0.78 | 9.82±0.96 |
| DS | 11.67±1.03 | 9.63±0.76 | 9.42±0.81 | 7.24±0.30 |
| **Tau** |  |  |  |  |
| Control | 145.10±9.03 | 134.57±3.62 | 137.35±5.21 | 135.67±8.45 |
| DS | 153.59±4.36 | 124.14±9.16 | 123.09±7.13 | 130.42±3.65 |
| **Pser** |  |  |  |  |
| Control | 0.06±0.00 | 0.06±0.01 | 0.05±0.01 | 0.05±0.01 |
| DS | 0.06±0.01 | 0.04±0.01 | 0.07±0.01 | 0.07±0.01 |
|  |  |  |  |  |
